# Lung Cancer Screening Eligibility and Screening Patterns Among Black and White Adults in the United States

**DOI:** 10.1001/jamanetworkopen.2021.30350

**Published:** 2021-10-20

**Authors:** Jessica W. Lozier, Stacey A. Fedewa, Robert A. Smith, Gerard A. Silvestri

**Affiliations:** 1 Division of Pulmonary Medicine, Thoracic Oncology Research Group, Hollings Cancer Center, Medical University of South Carolina, Charleston; 2 Surveillance and Health Equity Sciences, American Cancer Society, Atlanta, Georgia; 3 Prevention and Early Detection, American Cancer Society, Atlanta, Georgia

## Abstract

This cohort study compares the demographics of US adults who are currently being screened for lung cancer and assesses the potential association between proposed changes to the United States Preventive Services Task Force (USPSTF) screening recommendations and screening rates by race and age.

## Introduction

Lung cancer screening (LCS) with annual low radiation dose computed tomography (LDCT) for adults aged 55 years to 80 years with a 30 pack-year history of smoking was initially recommended by the United States Preventive Services Task Force (USPSTF) in 2013. In 2020, a large European trial demonstrated a mortality benefit from screening in persons aged 50 years with a 20 pack-year history of smoking.^1^ Based on these results and predictive modeling, the USPSTF recently updated their recommendations by lowering the age and pack-year requirements for screening from 55 years to 50 years and 30 pack-years to 20 pack-years, respectively.^2^

Reducing racial disparities in screening eligibility was an additional goal of the taskforce. Black individuals tend to develop lung cancer at a younger age with less pack-year history of smoking and have worse outcomes than White individuals.^3^ Thus, lowering the age and pack-year requirements will increase the number of Black persons eligible for LCS. We undertook this study to compare the demographics of US adults who are currently being screened for lung cancer to assess how the proposed changes might influence screening rates by race and age going forward.

## Methods

This cohort study followed the Strengthening the Reporting of Observational Studies in Epidemiology (STROBE) reporting guideline. This study was deemed exempt from institutional review board approval at the Medical University of South Carolina, and informed consent was waived because patients were deidentified in the survey from which the data were extracted.

We used 2017, 2018, and 2019 Behavioral Risk Factor Surveillance System (BRFSS) surveys from 10 states, 8 states, and 20 states, respectively, collecting data on detailed smoking history and LCS. Characteristics of respondents in these states were compared with the US population. The main outcome was the proportion of respondents who were eligible for LCS according to USPSTF criteria and self-reported receipt of LDCT in the past year according to race and ethnicity (non-Hispanic White respondents vs non-Hispanic Black respondents).

Respondents self-identified their race and ethnicity, and those who answered no to identifying as Hispanic and reported their race as Black or White were included. Race was considered in this study because of the disparities in lung cancer mortality between Black and White people and the new USPSTF guidelines were aimed at reducing these disparities. There were too few respondents who identified as Asian or Hispanic to examine race-specific screening characteristics.

Respondents meeting USPSTF criteria for LCS were selected (non-Hispanic White respondents: n = 13 132; non-Hispanic Black respondents: n = 518) and those who were missing data on LDCT data were excluded (253 non-Hispanic White respondents; 17 non-Hispanic Black respondents). The proportion of eligible individuals and those reporting screening and their demographic characteristics were computed according to race and/or ethnicity, with 95% CIs and χ^2^ tests (α = .05). All statistical analyses accounted for complex sampling design and were conducted with SAS statistical software, version 9.4 (SAS Institute). Statistical tests were 2-tailed, and data were collected from January 2017 to December 2019 and analyzed between January and July 2021.

## Results

This study included data from 13 380 US adults (non-Hispanic Black adults: 501 [3.7%]; non-Hispanic White adults: 12 879 [96.3%]). Among those eligible for screening, half currently smoked (non-Hispanic Black respondents: 51.1% [95% CI, 42.0%-60.1%]; non-Hispanic White respondents 50.9% [95% CI, 48.8%-53.0%]) and were aged 55 years to 64 years (non-Hispanic Black respondents: 50.4% [95% CI, 41.3%-59.4%]; non-Hispanic White respondents: 52.4% [95% CI 50.3%-54.5%]), and there were no significant differences according to race and ethnicity (Table 1). However, among those screened, more non-Hispanic Black respondents who were screened were aged 65 years to 80 years than non-Hispanic White respondents (47 of 66 [79.0%; 95% CI, 56.4%-91.6%] vs 1225 of 1988 [51.2%; 95% CI, 45.4%-57.0%]; *P* = 0.09). Of eligible non-Hispanic Black adults, 236 of 501 (34.2%; 95% CI, 26.9%-42.3%) were women, but women represented 35 of 66 (51.7%; 95% CI, 27.3%-75.1%) non-Hispanic Black adults who were screened. Conversely, the proportions of non-Hispanic White women who were eligible vs screened were similar (6055 of 12 879 [46.0%; 95% CI, 43.9%-48.2%] vs 957 of 1988 [46.3%; 95% CI, 40.6%-52.2%]). The rate of eligible adults reporting being screened was similar between racial groups (non-Hispanic Black adults: 16.5% [95% CI, 14.9%-18.2%]; non-Hispanic White adults: 14.0% [95% CI, 8.6%-21.9%,] *P* = 0.16). Most adults eligible for screening had a recent clinician visit (non-Hispanic Black respondents: 91.5% [95% CI, 86.1%-95.0%; non-Hispanic White respondents: 84.0% [95% CI, 82.5%-85.4%]).

**Table 1.** Demographic Characteristics, Smoking History, and Health Care Access of Respondents Who Were Eligible and Respondents Who Were Screened, BRFSS 2017-2019^a^

Characteristics	No. (%) [95% CI]					
	Respondents who were eligible			Respondents who were screened		
	Non-Hispanic White (n = 12 879)	Non-Hispanic Black (n = 501)	*P* value^b^	Non-Hispanic White (n = 1988)	Non-Hispanic Black (n = 66)	*P* value^b^
Smoking history						
Current	6833 (50.9) [48.8-53.0]	277 (51.1) [42.0-60.1]	.97	1132 (54.7) [48.9-60.4]	35 (52.6) [26.4-77.5]	.89
Former	6046 (49.) [47.0-51.2]	224 (48.9) [39.9-58.0]		856 (45.3) [39.7-51.1]	31 (47.4) [22.5-73.6]	
Sex						
Male	6819 (54.0) [51.9-56.1]	265 (65.8) [57.7-73.1]	.01	1029 (53.7) [47.8-59.4]	31 (48.4) [24.2-74.1]	.86
Female	6055 (46.0) [43.9-48.2]	236 (34.2) [26.9-42.3]		957 (46.3) [40.6-52.2]	35 (51.7) [27.3-75.1]	
Age, y						
55-64	5772 (52.4) [50.3-54.5]	231 (50.4) [41.3-59.4]	.64	763 (48.8) [43.0-54.6]	19 (21.1) [8.4-43.6]	.09
65-80	7107 (47.6) [45.5-49.7]	270 (49.6 [40.6-58.7])		1225 (51.2) [45.4-57.0]	47 (79.0) [56.4-91.6]	
Clinician visit in the past year	10 770 (84.0) [82.5-85.4]	451 (91.5 [86.1-95.0])	.001	1846 (94.8) [93.0-96.2]	64 (98.7) [94.2-99.7]	.064
Uninsured (55-64 y)	720 (11.9) [10.2-13.9]	231^c^		37 (3.3) [1.8-5.7]	NA^c^	
Proportion receiving LCS in the past year, % (95% CI)^d^	14.0 (8.6-21.9)	16.5 (14.9-18.2)				

Abbreviations: BRFSS, Behavioral Risk Factor Surveillance System; LCS, lung cancer screening; LDCT, low dose computed tomography; NA, not applicable.

^a^ Data for non-Hispanic White adults were missing on sex (n = 5), primary care clinician visit in the past year (n = 10); and insurance (n = 20).

^b^ *P* value based on χ^2^ statistic comparing non-Hispanic White adults vs non-Hispanic Black adults who were eligible and screened.

^c^ Unstable estimate.

^d^ Respondents were asked if they received LDCT for LCS the in past year. Respondents who answered yes were included in the numerator, people who responded no or reported LDCT for another reason were included in the denominator and were not considered to have LDCT in the past year.

## Discussion

The new USPSTF recommendations to screen younger patients with less smoking history will increase the number of Black persons who are eligible and should, in theory, decrease racial disparities in screening eligibility. However, the findings of this study suggest this may not be the case. While screening rates were similar between Black adults and White adults, nearly 80% of Black adults who reported being screened were of Medicare age, which suggests that access to care plays a significant role in whether someone is screened. While, screening data were only available in a limited number of states, however, respondent characteristics and smoking status in these states were similar to the US population (Table 2). Currently, Black adults are nearly twice as likely to be uninsured and more than twice as likely to have Medicaid, which may not cover LCS.^4^ Furthermore, studies show that screening rates for breast, cervical, and colorectal cancer are 41% to 47% lower among the individuals who are uninsured.^5^ Of the 8 million US adults who currently qualify for screening, estimates suggest that more than half those aged 50 years to 64 years have Medicaid or are uninsured.^6^ Limitations of this study included the small sample size, data were based on self-reports, and screening rates were higher than that reported nationally.

**Table 2.** Demographic Characteristics of the US Population and States Participating in BRFSS Lung Cancer Screening Module, 2017-2019

Characteristics	Respondents, No. (%) [95% CI]^a^	
	US total	States participating in LCS module^b^
Total	728 454	166 921
Smoking status		
Current	82 304 (12.8) [12.6-12.9]	18 795 (13.5) [13.1-14.0]
Every day	60 022 (9.2) [9.1-9.4]	13 806 (9.9) [9.5-10.3]
Some days	22 282 (3.5) [3.4-3.6]	4989 (3.6) [3.3-3.8]
Former	248 860 (35.3) [35.1-35.6]	57 141 (35.7) [35.1-36.3]
Never	369 513 (51.9) [51.7-52.2]	84 910 (50.8) [50.2-51.4]
US Region		
Northeast	137 002 (17.5) [17.3-17.6]	30 461 (16.8) [16.4-17.2]
Midwest	204 410 (21.6) [21.5-21.8]	41 352 (15.7) [15.5-15.9]
South	232 872 (38.2) [37.9-38.4]	72 610 (58.8) [58.3-59.3]
West	154 170 (22.7) [22.5-22.9]	22 498 (8.7) [8.5-8.9]
Male	309 619 (46.1) [45.8-46.4]	70 924 (46.1) [45.5-46.8]
Female	424 037 (53.9) [53.6-54.2]	95 910 (53.9) [53.2-54.5]
Age, y		
55-64	267 406 (44.2) [44.0-44.5]	59 636 (43.8) [43.2-44.4]
65-80	461 048 (55.8) [55.5-56.0]	107 285 (56.2) [55.6-56.9]
Race/ethnicity		
Non-Hispanic White	598 886 (73.6) [73.3-73.9]	141 283 (76.2) [75.5-76.9]
Non-Hispanic Black	52 146 (10.2) [10.0-10.3]	11 123 (11.2) [10.7-11.6]
Clinician visit in the past year	623 693 (86.9) [86.5-87.3]	144 276 (86.9) [86.5-87.3]
Uninsured, aged 55-64 y	21 178 (5.4) [5.3-5.5]	5051 (5.7) [5.4-6.1]

Abbreviations: BRFSS, Behavioral Risk Factor Surveillance System; LCS, lung cancer screening.

^a^ The following number of respondents were missing smoking status (US total, n = 27 777; module states, n = 6075); sex (US total, n = 698; module states, n = 87); race/ethnicity (US total, n = 15 010; module states, n = 3366); clinician visit (US total, n = 706; module states; n = 132); insurance (US total, n = 881; module states, n = 175).

^b^ Analyses were restricted to adults aged 55 years to 80 years. LCS data were not collected in all states. The following states participated in the BRFSS optional lung cancer screening module that collected detailed tobacco history and lung cancer screening questions in 2017 (n = 10 states): Florida, Georgia, Kansas, Maine, Maryland, Missouri, Nevada, Oklahoma, Vermont, and Wyoming; in 2018 (n = 8): Delaware, Maine, Maryland, New Jersey, Oklahoma, South Dakota, Texas, West Virginia; in 2019 (n = 20): Arizona, Idaho, Kansas, Kentucky, Maine, Maryland, Minnesota, Missouri, Montana, Nebraska, North Carolina, North Dakota, Oklahoma, Pennsylvania, Rhode Island, South Carolina, Utah, Vermont, West Virginia, and Wisconsin.

The new USPSTF recommendations will include screening eligibility for an additional 7 million younger Americans and a higher proportion of Black adults within this group will be uninsured. The findings here suggest that given the association insurance may have on the ability to be screened, disparities could paradoxically worsen rather than improve. Recognizing the role that social determinants of health care, including insurance, play in accessing screening services is essential to ameliorating disparities.
